# Editorial: Artificial intelligence in cardiac rhythmology

**DOI:** 10.3389/fcvm.2024.1466344

**Published:** 2024-08-05

**Authors:** Andrea Saglietto, Elena Cavallone, Michael Spartalis, Bert Vandenberk, Matteo Anselmino

**Affiliations:** ^1^ Division of Cardiology, Cardiovascular and Thoracic Department, “Citta Della Salute e Della Scienza” Hospital, Turin, Italy; ^2^ Department of Medical Sciences, University of Turin, Turin, Italy; ^3^ Department of Cardiology, National and Kapodistrian University of Athens, Athens, Greece; ^4^ Department of Cardiology, University Hospitals Leuven, Leuven, Belgium; ^5^ Department of Cardiovascular Sciences, KU Leuven, Leuven, Belgium

**Keywords:** artificial intelligence, electrophysiology, ECG, machine learning, deep learning

**Editorial on the Research Topic**
Artificial intelligence in cardiac rhythmology

Artificial Intelligence (AI) is revolutionizing the world of medicine, transforming both clinical practice and patient outcomes (1). In recent years, advancements in machine learning and big data analytics have driven AI's integration into various healthcare sectors. AI applications now range from real-time patient monitoring and predictive diagnostics to personalized treatment plans and robotic surgeries (2). This continuous evolution promises to enhance diagnostic accuracy, streamline clinical workflows and ultimately improve patient care.

In particular, AI has shown remarkable potential in cardiology, especially in electrocardiogram (ECG) analysis, as it can aid in early diagnosis, pattern recognition and risk stratification, paving the way for more responsive and personalized cardiovascular care (3). The growing number of publications on AI in healthcare underscores its increasing importance and the rising interest from the scientific community.

The application of AI in ECG diagnosis has seen significant advancements, with algorithms now capable of interpreting ECG data with high precision. AI-driven tools have been developed to detect arrhythmias, early acute myocardial infarction and other cardiac anomalies, as well as risk evaluations, more efficiently than traditional methods (4–6). These tools not only assist clinicians in making quicker decisions but also improve the accuracy of diagnoses. The progressive increase in AI use in ECG diagnostics enables continuous monitoring and early detection of life-threatening conditions, thereby enhancing preventive cardiology (7). The integration of AI in ECG reporting seems to allow quicker and more accurate interpretations, thus improving patient outcomes.

This Research Topic issue collects key innovations and studies that have enhanced ECG diagnostics through the diverse capabilities of AI-driven tools.

The extraction of beat-by-beat information from ECG is crucial for its diagnostic applications. Traditional methods are limited by their inability to generalize across diverse patterns, their time consumption and the difficulty in adapting to new or rare types of arrhythmias. Jimenez-Perez et al. made significant advancements in this field: they developed a synthetic data generation scheme to create diverse ECG traces and proposed novel segmentation-based loss functions to enhance prediction accuracy and segmentation precision across multiple ECG databases. Despite some limitations, such as data representation constraints and computational challenges, the work provides a substantial advancement in ECG analysis and highlights the need for standardized metrics and open-access resources.

A convolutional neural network (CNN) using a single-lead ECG (D1) achieved satisfactory performance compared to the standard 12-lead framework in predicting cardiac abnormalities (area under the curve—AUC—difference 8.7%), with further precision when adding a second lead (Saglietto et al.). These findings underscore the potential for simpler ECG setups to be effectively used in AI-driven cardiac analysis, making routine diagnostics more accessible.

Building on the promise of CNN models, another study (Chang et al.) developed a machine learning model using a twelve-lead ECG to predict acute mortality risk in the emergency department. High accuracy was demonstrated in predicting 30-day mortality across various diseases (AUC 0.84), proving to be an invaluable screening tool that complements traditional early warning scores. The model also had good results in predicting one-year mortality. This approach not only enhances the efficiency of AI in the emergency care but also provides a robust method for early risk stratification, enabling timely interventions.

An AI model based on 8-lead electrocardiograms was trained to detect atrial septal defect in adults (Luo et al.), showing promising results in identifying patients with high accuracy, precision, recall and specificity (AUC 0.99), again highlighting the applicability of a lower number of leads, as with portable devices.

Another significant application of AI in interpreting ECG is the detection of arrhythmias. Chen et al. employed wavelet time-frequency maps and the Swin Transformer deep learning model for this purpose. By using a large ECG dataset and removing noise, the model captured subtle changes in ECG signals, crucial for early diagnosis, especially in asymptomatic patients. The model achieved high classification accuracies (99.34% intra-patient and 98.37% inter-patient). The Swin Transformer's advanced self-attention mechanism overcame traditional limitations, enhancing diagnostic speed and accuracy and provided a comprehensive view of arrhythmias by analyzing ECG data across multiple time and frequency scales.

Bocanegra-Pérez et al. focused on standardizing the diagnosis of ventricular arrhythmias and the identification of the precise site of origin through machine learning. They tested three different AI models, developing an approach that integrates QRS complex morphology with clinical variables, and achieved promising results in distinguishing between right and left ventricular outflow tract arrhythmias, demonstrating high accuracy and sensitivity. Additionally, the study explored unsupervised analysis to identify clustering patterns related to specific arrhythmia origins.

In conclusion, the integration of AI in cardiology, particularly in ECG analysis, holds immense promise. From early diagnosis to predictive analytics, AI is set to transform cardiac care, making it more efficient, accurate and personalized. The progressive adoption of AI technologies in cardiology will undoubtedly lead to better patient outcomes and a more proactive approach. The continuous advancements in AI-driven tools and their applications in various aspects of cardiology exemplify the profound impact of technology on modern medicine, heralding a new era of healthcare innovation.
